# Effects of Heat Shock on Resistance to Parasitoids and on Life History Traits in an Aphid/Endosymbiont System

**DOI:** 10.1371/journal.pone.0075966

**Published:** 2013-10-15

**Authors:** Luis Cayetano, Christoph Vorburger

**Affiliations:** 1 Institute of Integrative Biology, ETH Zürich, Zürich, Switzerland; 2 EAWAG, Swiss Federal Institute of Aquatic Science and Technology, Dübendorf, Switzerland; University of Poitiers, France

## Abstract

Temperature variation is an important factor determining the outcomes of interspecific interactions, including those involving hosts and parasites. This can apply to variation in average temperature or to relatively short but intense bouts of extreme temperature. We investigated the effect of heat shock on the ability of aphids (*Aphis fabae*) harbouring protective facultative endosymbionts (*Hamiltonella defensa*) to resist parasitism by Hymenopteran parasitoids (*Lysiphlebus fabarum*). Furthermore, we investigated whether heat shocks can modify previously observed genotype-by-genotype (*G x G*) interactions between different endosymbiont isolates and parasitoid genotypes. Lines of genetically identical aphids possessing different isolates of *H. defensa* were exposed to one of two heat shock regimes (35°C and 39°C) or to a control temperature (20°C) before exposure to three different asexual lines of the parasitoids. We observed strong *G x G* interactions on parasitism rates, reflecting the known genetic specificity of symbiont-conferred resistance, and we observed a significant *G x G x E* interaction induced by heat shocks. However, this three-way interaction was mainly driven by the more extreme heat shock (39°C), which had devastating effects on aphid lifespan and reproduction. Restricting the analysis to the more realistic heat shock of 35°C, the *G x G x E* interaction was weaker (albeit still significant), and it did not lead to any reversals of the aphid lines' susceptibility rankings to different parasitoids. Thus, under conditions feasibly encountered in the field, the relative fitness of different parasitoid genotypes on hosts protected by particular symbiont strains remains mostly uncomplicated by heat stress, which should simplify biological control programs dealing with this system.

## Introduction

Open-ended cycles of coevolution can result from genotype-by-genotype (*G x G*) interactions between parasites and hosts, with regular turnover of certain alleles and their corresponding phenotypes through time [1], [2]. These coevolutionary cycles are maintained by negative frequency dependent selection [3]–[6]. *G x G* interactions between interacting lineages can be modified by biotic and abiotic variation [7]–[12]. This potentially makes the outcomes of these interactions more complex and/or unpredictable than the simpler alternatives of recurring oscillations of certain allele frequencies over time, or the convergence upon stable mean frequencies of certain alleles [8], [11], [12]. This element of complexity/unpredictability can also pose a challenge to biocontrol strategies utilizing pathogens or parasites of pests [13].

By interacting with host and parasite genotypes, variation in factors such as nutritional quality [14] and the presence of other organisms [7] may be manifested as a genotype-by-genotype-by-environment (*G x G x E*) interaction and thus change the spatial and temporal nature of reciprocal selection. Temperature variation is perhaps the most ubiquitous factor facing organisms, and it is known to be important in determining the outcomes of various host-parasite interactions [8], [15], [16]. More specifically, it can alter aspects of host immunology or parasite/pathogen virulence, and favour either party depending upon the particular system [8], [15]–[20].

Heat shock - relatively short bouts of high temperature - can also have important effects on the outcomes of host-parasite interactions [21]. Aphids, their endosymbionts and parasitoid wasps - the study system in the current investigation - are known to be affected by heat shock in various life-history stages. These effects include a strongly deleterious effect on reproduction in the pea aphid *Acyrthosiphon pisum* Harris (Hemiptera: Aphididae) via the undermining of the regular function of the obligate aphid endosymbiont *Buchnera aphidicola* [22], [23] and a reduction in the ability of the facultative endosymbiont *Hamiltonella defensa* to confer resistance to this aphid against the parasitoid *Aphidius ervi* [24]. *Hamiltonella defensa* can also mitigate the negative effects of heat shock on survivability [17], [21], [24], pointing to the versatility of these bacteria. Clearly, heat shock has the potential to complicate the interactions between Hymenopteran parasitoids and the endosymbiont-protected aphids they target. Given the importance of temperature for various aspects of the ecology of this system, as well as the evident strength of *G x G* interactions between the parasitoids and the endosymbionts protecting their hosts [25], [26], it is worth asking whether a more holistic view of these interactions can be gleaned by noting whether they are modified by heat shock [8], [13]. We addressed this question by using the relatively well-understood study system consisting of the black bean aphid *Aphis fabae* (Hemiptera: Aphididae), its secondary (non-obligate) bacterial endosymbiont *Hamiltonella defensa*, and the primary Hymenopteran parasitoid of *A. fabae*, *Lysiphblebus fabarum* (Hymenoptera: Braconidae, Aphidiinae).

*Hamiltonella defensa* confers significantly improved resistance to *A. fabae* against parasitoid wasps [25], [27], a situation originally observed in pea aphids [28]. This presence augments the general and weak protection conferred by the aphid's immune system [29] with a defense that, depending on the particular strain of *H. defensa* and the parasitoid it interacts with, can range from providing no advantage compared to the defenses already present in aphids, to providing a greatly increased defense [25]. The bacterium can thus mediate the specificity of the interaction between parasitoids and hosts, which translates into significant host line x parasitoid line interactions, albeit from a *G x G* interaction involving the endosymbiont rather than the host. Following on from a similar investigation that looked at these interactions in the context of average temperature variation [26], we further extend the study of *G x G x E* interactions in the context of abiotic factors and protective endosymbionts. The experiment reported here differs in that it considers the efficacy of heat shock in changing *G x G* interactions. We used an experimental setup in which aphid sublines comprised of genetically identical individuals harbouring different isolates of *H. defensa* were first exposed to one of two heat shock treatments (35°C and 39°C) or a control treatment (20°C), and then attacked by parasitoids of different genotypes known to differ in their ability to defeat the resistance conferred by the bacteria. Further we addressed whether the effects of heat shocks on aphid life-history traits were modified by the symbionts. We detected a significant *G x G x E* interaction on parasitism rates, but it was mainly due to the more severe heat shock of 39°C, which was fatal to a large proportion of aphids and thus of limited relevance. The *G x G x E* interaction was much weaker (albeit still marginally significant) when just considering the control treatment and the 35°C heat shock (representing a more ecologically realistic range), without any changes in susceptibility rankings. Thus, genotype-by-genotype specificity in this system is relatively robust to bouts of hot temperature as they are naturally encountered. We further show that heat shock effects on aphid lifetime reproduction are modified by the symbionts.

## Methods

### Study system

The black bean aphid, *Aphis fabae* is an aphid species that reproduces through cyclical parthenogenesis and is widely distributed across temperate regions of the northern hemisphere. It is also known as a crop pest [30]. It can be reared in the laboratory on broad beans plants (*Vicia faba*), which is one of its preferred secondary host plants (used during its asexual stage during spring and summer), and the aphid's asexual mode of reproduction can be maintained when clones are kept under summer-like conditions (20°C with a 16 h photoperiod).

This aphid is targeted by several Hymenopteran parasitoids, the most important of which is *Lysiphlebus fabarum* (Hymenoptera: Braconidae, Aphidiinae) [31]. This koinobiont and mostly thelytokous [32], [33] wasp oviposits in individuals of several aphid species at any of their life history stages, with one egg administered per sting. The developing larva eventually kills its host and emerges from the host's desiccated remains, or ‘mummy’, after undergoing pupation.

The bacterium *Hamiltonella defensa* of the Enterobacteriaceae [34] can be harboured by several species of aphid and is transmitted vertically with high fidelity. Much less frequently it can also be transmitted horizontally, either via the contaminated ovipositors of parasitoid wasps [35] or through sex [36]. The symbiont provides protection from the parasitoid *L. fabarum* in *A. fabae* and from *Aphidius eadyi* and *Aphidius ervi* in the pea aphid *Acyrthosiphon pisum* [27], [28], [37], [38]. The precise mechanisms pertaining to this ability have not been fully elucidated, but toxins encoded by bacteriophages known collectively as *Acyrthosiphon pisum secondary endosymbionts* (APSE), which are integrated into the genomes of *H. defensa*, appear to be crucial [39]–[41]. The bacterium, in spite of its demonstrated benefits and its ability to be transmitted via at least three routes, evidently does not reach fixation in wild populations of *A. fabae*. This assessment was gauged by the fact that *H. defensa* was found in just over half of more than 400 individuals in a wide-ranging collection drawing from different localities in France and Switzerland (R. Rouchet, J. Herzog & C. Vorburger, unpublished data). This moderate frequency may result from the costs borne by the aphids carrying the bacterium when parasitoids are not present [42], [43].

### Experimental lines

We utilized asexual lines of the parasitoids and hosts. By having a uniform host genetic background, the effects of different endosymbiont strains can be measured unconfounded by underlying genetic variation, and by using asexual lines of parasitoids as well, the exact same combinations of hosts and parasitoids can be replicated, providing the necessary power to test for genetic interactions. Our experiment used four sublines of a single clone of *A. fabae fabae*, the nominal subspecies of *A. fabae*. This clone (nr. 407) was collected in July 2006 in St. Margrethen, Switzerland, and has been confirmed to be free of any known facultative symbionts of aphids [27]. It was chosen for the current investigation because it was found in a previous study to be highly susceptible to parasitoids when lacking endosymbionts [27]. The other three sublines were each infected with a different isolate of *H. defensa*, acquired from the hemolymph of donor clones via microinjections as explained in Vorburger et al. 2010 [44]. The injections had been performed more than 50 generations before the sublines' use in the current study. These led to stable, heritable symbiotic associations that were corroborated by diagnostic PCR just prior to the experiment. The infected sublines are labeled as 407^H76^, 407^H323^ and 407^H402^, with the superscripts denoting the specific *H. defensa* isolates. These isolates were chosen because of their wide range of efficacy in protecting against different genotypes of *L. fabarum*. [25], [45]. Notably, a comparison of two endosymbiont gene fragments – *accD* (acetyl-CoA carboxylase, 374 bp) and *murE* (Murein, 474 bp) – found two distinct haplotypes present, one corresponding to H76 and the other corresponding to H323 and H402, with a sequence divergence of 0.94% (J-C. Simon, unpublished data). The clone from which H402 was originally obtained was collected in St. Margrethen, Switzerland, on 1 July 2006, while the clones from which isolates H76 and H323 were obtained were collected in La Grande Motte, France, on 17 May 2006 and in Aesch, Switzerland, on 27 June 2006, respectively. Due to aphids' propensity for dispersal [46] and a population genetic survey revealing low rates of genetic differentiation throughout Europe for this species [47], the clones from which the isolates were acquired cannot be assumed to originate from truly separate populations.

The parasitoids used in this study came from three asexual lines of *L. fabarum*. These were designated as 06-15, 07-64 and 09-369, and were chosen on the basis of their differential ability to parasitize particular sublines of *A. fabae* [25], [45]. They were originally obtained from *A. f. fabae* in the wild, making them appropriate for this study because of their evident adaptation to these aphids in a natural setting. 06-15 was obtained from Sarzana, Italy, in 2006, 07-64 from Wildberg, Switzerland in 2007, and 09-369 from Orbe, Switzerland in 2009. Thelytokous lines of *L. fabarum* are able to restore diploidy with central fusion automixis, thereby losing heterozygosity. Our wasps are thus largely genetically constant within lines, though they lack true clonality. Adult wasps emerge at about two weeks after oviposition.

### Experimental design

#### Experiment 1: effects of heat shock on resistance against parasitoids

**Figure 1:**
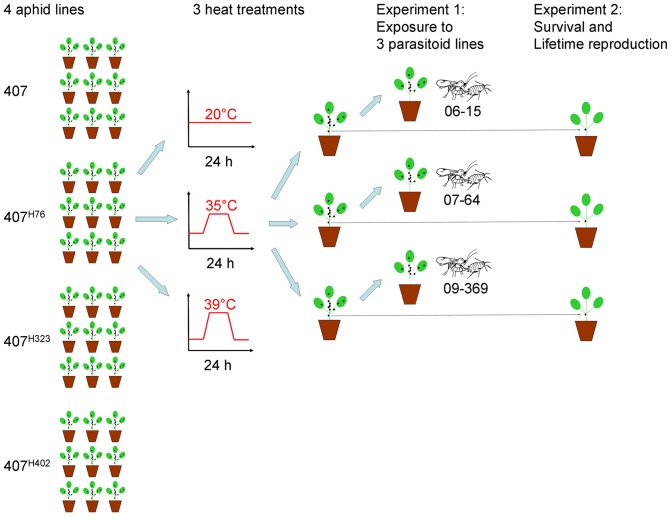
Illustration of the procedures for one block of the experiment. The entire experiment comprised six complete blocks, with 216 replicates in total. Blocks consisted of plastic trays with replicates assigned to random positions within these trays. They were temporally staggered such that three blocks were processed per day over two consecutive days.

This experiment compared the resistance of the four aphid sublines against the three asexual lines of *L. fabarum* under three treatments: two heat shock regimes (35°C and 39°C) and one control treatment (20°C). Each of the aphid sublines were exposed to one of three thermal regimes and to one of three parasitoid lines for a full factorial design. Six complete randomized blocks were established, each containing 36 replicates (4 sublines x 3 treatments x 3 parasitoid lines; Fig.1). The aphid sublines were split up into the required number of replicates and propagated at 20°C for two generations before the start of the experimental treatments to exclude between-line variation related to environmental maternal or grandmaternal effects carried over from the stock culture. For each generation, fresh seedlings of *Vicia faba* (broad beans) were grown in 0.07 litre plastic pots. To produce the test generation, three adult females of the previous generation were placed together on a new seedling to reproduce and then removed after 24 hours. Replicates were assorted randomly in each block. The offspring were kept at 20°C in an environmental chamber and left to grow for another 48 hours to their second or third instar stages. They were then moved to their respective heat treatments in three identical computer-controlled growth cabinets (Percival E-36L; Percival Scientific Inc.; Perry USA) running on a 16 hour photoperiod for 24 hours. To control for possible differences in the developmental timing of aphids, the average temperature experienced by all treatments over the 24 hours of temperature manipulation was 20°C. This was achieved by exposing the aphid colonies in the two heat shock treatments to steadily rising temperature over a period of two hours, followed by constant peak temperature for four hours, and a gradual decline over another two hours to a minimum of 13.4°C (for the 39°C treatment) and 15°C (for the 35°C treatment), with the appropriate thermal increments and decrements corresponding to calculated heat curves. The minimum temperatures were maintained for eight hours before the climb to peak temperatures and for eight hours after the decline from them. Due to the large number of replicates, temperature treatments were administered over two days, with three blocks processed on each day. The surviving aphids were counted after removal from the growth cabinets, and each replicate aphid colony was then exposed to two wasps for six hours in the original environmental chamber, again at 20°C. The counts showed that a 39°C heat shock is very severe and virtually at the lethal limit of *A. fabae*. We lost a total of 27 replicates (out of 216 cages in total) because no aphids survived the 39°C heat shock in these cages, and the remaining replicates contained much lower numbers of survivors on average than those of the other two treatments (control treatment: 29.36±7.14 SD; 35°C heat shock: 29.58±6.71; 39°C heat shock: 12.04±8.74; Tukey's test: *P* = 0.982 for control vs. 35°C, *P*<0.001 for control/35°C vs. 39°C). Six replicates exposed to the 39°C heat shock had just a single survivor, which was used for experiment 2 (see below), leading to the loss of another 6 replicates for experiment 1.

After the wasps were removed again from the cages, the colonies were kept in the chamber at this temperature until successfully parasitized aphids turned into mummies. The mummies were counted ten days after exposure to parasitoids.

Aphids were exposed to the wasps after and not during the heat shock because doing so would potentially have confounded the effects of temperature on host resistance with temperature-induced changes in wasp behavior (if they had survived the heat shock at all). Our design tested specifically whether experiencing a heat shock (and its potential effects on defensive symbionts) affected the ability to resist parasitism subsequent to the heat shock.

#### Experiment 2: effects of heat shock on life history traits

To test for the effects of heat shock on life-history traits of aphids, a single individual was removed from each cage before the aphid nymphs that survived the heat shock were counted and exposed to parasitoids. These individuals were placed individually on a new caged seedling and allowed to grow to adulthood (Fig. 1). Survival was first checked three days after the heat shock and every second day thereafter, as well as on transfer days (see below). Cages in which the focal individual could not be found were rechecked on the following day to ensure that the individual had not been overlooked. If the individual could not be found on the second inspection either, it was presumed to have died the previous day. For measuring the aphids' lifetime reproduction, the focal individuals were repeatedly transferred to a new plant to allow counting the number of offspring produced on the old plant. Transfers happened every five days initially and at somewhat longer intervals when the remaining aphids became old and reproduction decreased. Unfortunately, this led to several handling errors in the late phase of the experiment because offspring that had already turned adult were mistaken for the focal individual and transferred to the new plant. This became immediately evident as individuals that had already ceased to reproduce appeared to pick up reproduction again at a high rate, which does not occur naturally in aphids. In these cases it was still possible to determine the lifetime reproduction (the number of offspring produced until reproduction seized), but the longevity of the focal individuals had to be treated as missing data. One replicate was lost because the focal individual was accidentally killed by the experimenter, and 27 replicates from the 39°C heat shock treatment were missing because no individuals had survived the heat exposure (see above). This explains why the residual df in the analyses are lower than would be expected from the experimental design (Fig. 1).

### Statistical analyses

Our measure of parasitism success was the proportion of aphids exposed to wasps that were successfully parasitized, i.e. mummified. Severe overdispersion prevented the use of a generalized linear model with binomial errors for these proportion data. Therefore, we resorted to using a linear model on arcsin-square root transformed proportions. The transformation improved the error distribution, but it could not achieve a normal distribution of residuals (K-S test, *P*<0.001), nor could it fully amend the heterogeneity of variances (Levene's test, *F*_35, 146_ = 4.71, *P*<0.001) due to the very low variances in aphid subline x parasitoid line combinations that showed complete or near-complete resistance. Although linear models are relatively robust to deviations from parametric assumptions [48], these constraints required very cautious interpretation of marginal p-values. We tested for the effects of aphid subline, parasitoid line and heat shock treatment (all fixed) as well as their interactions. The variance among blocks was pooled into the residual term of the final analysis, as its effect was far from significant (*P* = 0.357). Due to imbalances in the design resulting from lost replicates in the 39°C heat shock treatment (see above) we used type III SS. After the analysis including all aphid sublines, we repeated the analysis restricted to three *H. defensa*-infected sublines, because only in this case is the aphid subline x parasitoid line interaction strictly interpretable as a *G x G* interaction between the parasitoids and the hosts' symbionts.

The life history responses to heat shock (time survived after heat shock and the lifetime number of offspring produced) were also analyzed with linear models and type III SS. We tested for the effects of block, aphid subline, heat shock treatment and the subline x treatment interaction. Again we ran the analyses once for all aphid sublines and once restricted to the *H. defensa*-infected sublines, because only in the latter case can the subline x treatment interaction strictly be interpreted as a *G x E* interaction between symbiont isolates and the occurrence of heat shocks.

Data were analysed using SPSS/PASW 18.

## Results

### Experiment 1

**Figure 2:**
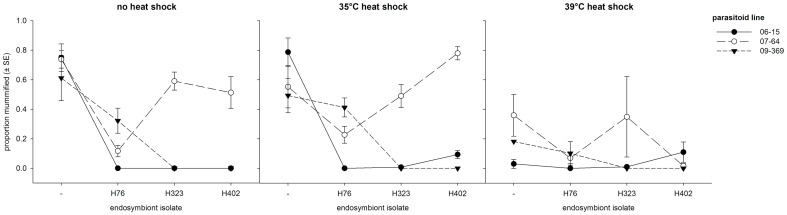
Genotype-by-genotype interactions across three temperature treatments. Vertical axis is proportion of mummified aphids (raw values shown here). Horizontal axis denotes endosymbiont isolates/aphid sublines; - denotes aphid subline lacking symbiont. Vertical bars are standard errors.

When all aphid sublines were included in the analysis (left side of Table 1), aphid subline, parasitoid line and treatment all had significant effects on the proportion of aphids parasitized (Table 1; Fig. 2. Fig. 2 is shown with raw values to assist visual inspection, but general patterns are unchanged compared to transformed data). Unsurprisingly, the *Hamiltonella-free* aphids suffered the highest rates of parasitism overall, because they were susceptible to all parasitoid lines, of which 07-64 was the most infective when averaged across the different host sublines and treatments (Fig. 2). We also observed a significant aphid subline x treatment interaction and a very strong aphid subline x parasitoid line interaction, the latter reflecting that the sublines harbouring *H. defensa* showed complete or partial resistance to some (but not all) of the parasitoid lines. The main effect of treatment was mainly caused by the much lower rates of parasitism after the 39°C heat shock (Fig. 2), yet this was largely due to the fact that this temperature was virtually at the lethal limit for *A. fabae*. The aphid colonies were decimated by the heat shock already before exposure to parasitoids (see Methods), and many individuals died soon after exposure to wasps (see Experiment 2 below), providing insufficient time for mummies to be formed in many cases. Those results that still could be obtained from the 39°C heat shock treatment showed marked differences in the susceptibility rankings of aphid sublines to parasitoid lines compared to the other two treatments (Fig. 2), which resulted in a significant aphid subline x parasitoid line x treatment interaction (Table 1). However, this *G x G x E* interaction should be interpreted cautiously given the high mortality in the most extreme treatment. Indeed, when the analysis excluded the 39°C heat shock treatment, neither the main effect of temperature nor the three-way interaction remained significant (Table 1).

**Table 1:** General linear model results for the proportion of aphids parasitized (arcsin-square root transformed).

	all aphid sublines				*Hamiltonella*-infected sublines only			
Source	Df	MS	*F*	*P*	df	MS	*F*	*P*
(a) all treatments								
Aphid subline	3	2.106	44.807	<0.001	2	0.005	0.214	0.808
Parasitoid line	2	2.483	52.832	<0.001	2	2.974	132.603	<0.001
Treatment	2	1.069	22.738	<0.001	2	0.461	20.545	<0.001
Aphid subline x parasitoid line	6	0.816	17.365	<0.001	4	0.987	44.035	<0.001
Aphid subline x treatment	6	0.196	4.175	0.001	4	0.087	3.879	0.005
Parasitoid line x treatment	4	0.073	1.554	0.190	4	0.219	9.773	<0.001
Aphid subl. x parasitoid l. x treat.	12	0.209	4.443	<0.001	8	0.145	6.471	<0.001
Residual	147	0.047			113	0.022		
(b) 39° heat shock excluded								
Aphid subline	3	3.777	77.613	<0.001	2	0.063	3.364	0.039
Parasitoid line	2	3.075	63.174	<0.001	2	4.379	233.208	<0.001
Treatment	1	0.077	1.583	0.211	1	0.208	11.074	0.001
Aphid subline x parasitoid line	6	1.398	28.720	<0.001	4	1.332	70.912	<0.001
Aphid subline x treatment	3	0.132	2.715	0.048	2	0.105	5.602	0.005
Parasitoid line x treatment	2	0.039	0.796	0.454	2	0.020	1.071	0.347
Aphid subl. x parasitoid l. x treat.	6	0.050	1.035	0.406	4	0.052	2.745	0.033
Residual	120	0.049			90	0.019		

Separate analyses are presented for all three treatments (a) and for the control and 35°C heat shock treatments only (b), because the high mortality after the 39°C heat shock rendered those results less reliable. The analyses were conducted once for all aphid sublines and once restricted to the *Hamiltonella defensa*-infected sublines, because only in the latter case does the aphid subline x parasitoid line interaction strictly reflect the *G x G* interaction between the parasitoids and the hosts' symbionts.

To enable a clear assessment of the variation due to the different isolates of *H. defensa*, we repeated the analyses for the *H. defensa*-bearing aphid sublines only (Table 1, right side). The results were very similar except for the main effect of aphid subline, which was no longer significant without the most susceptible, *H. defensa*-free aphids, and the parasitoid line x treatment interaction now exhibiting significant variation. The aphid subline x parasitoid line interaction remained highly significant (Table 1), confirming that the *G x G* interaction between symbionts and parasitoids has a strong influence on the outcome of parasitoid attacks. The three-way (*G x G x E*) interaction remained significant as well (Table 1). Because the high mortality after the 39°C heat shock might have led to spurious results, we also repeated this analysis for the control treatment and the 35°C heat shock only. Indeed, the parasitoid line x treatment interaction was no longer significant in the restricted analysis, but the three-way interaction remained significant, albeit only marginally so (*P* = 0.033, Table 1). Inspection of Figure 2 suggests that this interaction is largely due to the aphid subline harbouring *H. defensa* isolate 402 becoming more susceptible to two of the three parasitoid lines after a 35°C heat shock, but this did not upturn the susceptibility rankings of host sublines to the different parasitoid genotypes (Fig. 2).

### Experiment 2

**Figure 3:**
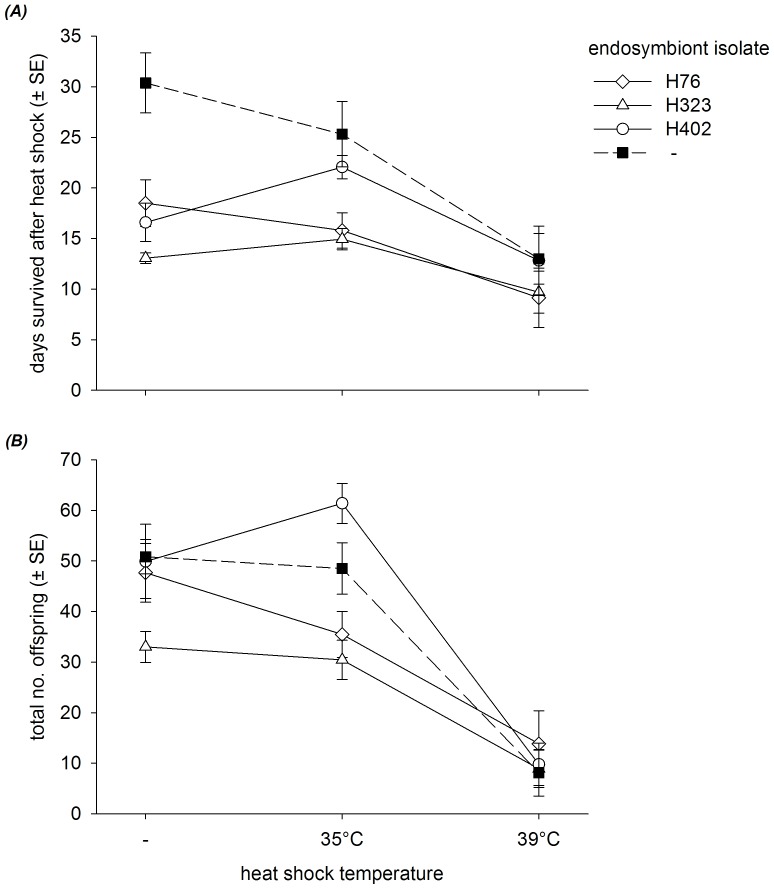
Genotype by environment interactions between endosymbionts and temperature with respect to aphid life history. Horizontal axes denote aphid/endosymbiont isolate sublines; - denotes aphid subline lacking symbiont. Vertical bars are standard errors. (***A***). Number of days survived after heat shock treatment. (***B***). Total number of offspring produced.

The 39°C heat shock treatment had drastic effects on the survival of aphids. In a large fraction of replicates, all individuals succumbed to the heat shock (see Methods), precluding their use in the experiment comparing life-history traits. The survivors that could be used in the experiment exhibited strongly reduced lifespans after the heat shock compared to individuals from the other two treatments (Fig. 3A), which resulted in a significant treatment effect in the analysis (Table 2). Longevity also differed significantly among aphid sublines (Table 2); the subline without *H. defensa* had the longest lifespan overall (Fig. 3A). The aphid subline x treatment interaction across all aphid sublines was not significant, and it remained (marginally) non-significant when the analysis was restricted to sublines harbouring *H. defensa*. Nevertheless, it was notable that the subline possessing *H. defensa* isolate 402 showed a substantial increase in lifespan of about 5 days after the 35°C heat shock compared to the control treatment (Fig. 3A).

**Table 2:** General linear model results for (a) the number of days aphids survived after the temperature manipulation (20°C control, 35°C and 39°C heat shocks) and for (b) the lifetime number of offspring they produced after these treatments.

	all aphid sublines				*Hamiltonella*-infected sublines only			
Source	df	MS	*F*	*P*	df	MS	*F*	*P*
(a) Days survived after heat shock								
Block	5	135.052	1.949	0.089	5	128.990	2.319	0.047
Aphid subline	3	696.135	10.046	<0.001	2	252.594	4.540	0.013
Treatment	2	1258.146	18.156	<0.001	2	609.879	10.963	<0.001
Aphid subline x treatment	6	176.627	2.549	0.022	4	92.640	1.665	0.162
Residual	158	69.295			124	55.632		
(b) Lifetime number of offspring								
Block	5	1077.368	2.893	0.016	5	979.951	2.473	0.036
Aphid subline	3	2015.914	5.414	0.002	2	2978.658	7.517	0.001
Treatment	2	21034.120	56.489	<0.001	2	15034.513	37.939	<0.001
Aphid subline x treatment	6	984.981	2.645	0.018	4	1292.439	3.261	0.014
Residual	171	372.355			130	396.276		

The analyses were conducted once for all aphid sublines and once restricted to the *Hamiltonella defensa*-infected sublines, because only in the latter case can the subline x treatment interaction strictly be interpreted as a *G x E* interaction pertaining to different symbiont isolates.

The differences in longevity among treatments translated into significant differences in lifetime reproduction (Table 2), with only 10.56 offspring produced on average after the 39°C heat shock compared to 45.35 and 44.07 offspring in the control and 35°C heat shock treatments, respectively (Fig. 3B). There was also significant variation among aphid sublines and a significant subline x treatment interaction, which appeared to be due to the only case in which the presence of the symbiont corresponded to an advantage over the *Hamiltonella*-free subline in terms of life history measures, namely the increase in lifetime reproduction after a 35°C heat shock for subline 407^H402^ (Fig. 3B). However, this interaction remained significant when the analysis was restricted to the protected sublines only (Table 2), indicating that the different isolates of *H. defensa* responded unequally to the heat shocks, which in turn affected the aphids' life-history traits.

## Discussion

This study recovered the strong *G x G* interaction between endosymbionts and parasitoids on the outcome of parasitoid attacks that has become a hallmark of this system [25], [26], [45]. Here we tested whether this specificity of symbiont-conferred resistance to parasitoids is modified by bouts of high temperature, i.e. whether there is a *G x G x E* interaction induced by heat shocks. The result we obtained is not straightforward. Although the *G x G x E* interaction was highly significant when all treatments were considered, it was mainly driven by the most extreme treatment of a 39°C heat shock. As shown by the high mortality and strongly reduced reproduction of aphids submitted to this treatment, 39°C is at the lethal limit of *A. fabae*, making it highly questionable whether this interaction is of any biological relevance in the field, especially since such temperatures are reached only very rarely in central Europe (see below). However, there was still a marginally significant *G x G x E* interaction when just the control and the 35°C heat shock treatment were considered for the three aphid sublines possessing *H. defensa*. This indicates that the different isolates of *H. defensa* varied somewhat in their response to heat shocks, which then translated into minor shifts in the relative susceptibilities of the protected aphid sublines to the different parasitoid genotypes. But note that the susceptibility rankings were in no case reversed by the 35°C heat shock. In that sense, the outcomes resulting from heat shock are not markedly different from average temperature manipulations on the same system [26], at least within the range of conditions that *A. fabae* is most likely to encounter in the wild. Temperatures of 35°C are realistic but relatively uncommon in central Europe; according to data collated over a period of 20 years from 1993 to the present across 37 Swiss sites situated at<600 metres of elevation above sea level that have experienced maximums of at least 35°C at some point during this time span, temperatures of or greater than 35°C occurred on average in two separate years for each site, and occurred on average on about 8 days across these years for each site (or about four times for each year that experienced this temperature). Temperatures of 39°C or over were experienced only four times at all, for the entire 20 year span, with each occurrence happening only once in each respective site (data collated by MeteoSwiss and analysed with CLIMAP-net 8.3; http://www.meteosuisse.admin.ch/web/en/services/data_portal/climap-net.html). Furthermore, the ecological relevance of these temperatures would still critically depend upon the geographical range across which they are experienced at a given time, as well as the degree of dispersal of aphids from adjacent localities that did not experience such an intense heat spike. These would presumably have the effect of further diluting the efficacy of temperature extremes in determining the outcomes of these interactions.

The susceptibility of aphids to the parasitoid (measured as the proportion of aphids exposed to wasps that were mummified) was notably lower after the 39°C heat shock. At first glance, this is opposed to findings by Bensadia et al. 2006 [17] and Guay et al. 2009 [24], who found that heat stress reduces the protection by *H. defensa* against parasitoids in pea aphids. But our result is certainly not indicative of increased protection under heat stress by the presence of *H. defensa*. As evidenced by the high death rate and the reduced longevity of survivors from the 39°C heat shock treatment, this was instead likely due to the aphids' poor condition as a result of the heat shock. This presumably rendered the aphids unsuitable as hosts for parasitoids even if the parasitoids could overcome host resistance mechanisms and initiate the early stages of ontogeny, because most aphids died before the parasitoids could complete their development. Alternatively, the poor condition of aphids after exposure to this temperature could have manifested itself in lower parasitism rates due to parasitoid behavior. Wasps may have avoided ovipositing into aphids after assessing their poor quality as hosts. We cannot exclude this possibility, although we believe that the moribund state of aphids after the 39°C heat shock was the main reason why so few mummies were formed after this treatment.

The 39°C heat shock in our study was more extreme than any treatments applied in the study by Guay et al. (2009), who reported a substantial reduction of *H. defensa*-conferred resistance in response to heat shocks. On the other hand, the 35°C heat shock over 4 h was comparable, although applied only once in the present case as opposed to once daily [24]. We also observed some weakening of the protection by *H. defensa* after aphid exposure to 35°C, albeit not universally so. For example, aphid sublines 407^H323^ and 407^H402^ were completely resistant to parasitoid lines 06-15 and 09-369 in the control treatment, and this resistance was largely retained after the 35°C heat shock except for subline 407^H402^, which became partially susceptible to parasitoid line 06-15 (Fig. 2). Aphid subline 407^H76^, which in the control treatment was completely resistant to parasitoid line 06-15 but only partially protected against 07-64 and 09-369, remained resistant to 06-15 after the 35°C heat shock but became even more susceptible to the other two lines (Fig. 2). The effect of the heat shock on protection by *H. defensa* was thus limited overall and depended on the symbiont isolate and parasitoid line involved, which was ultimately responsible for the marginally significant *G x G x E* interaction we observed.

The mechanism by which the overall susceptibility of *Hamiltonella*-free sublines after exposure to 35°C as compared to the control regime increased is not clear, though one possibility is a negative effect on the density of *H. defensa*. This is made plausible by known effects of heat on the densities of other bacterial taxa. For example, *Wolbachia*, the most abundant bacterial symbionts of insects, can be ‘cured’ with high temperature [49], while the abundance of the obligate endosymbiont of aphids, *Buchnera aphidicola*, is known to exhibit a reduction after short periods of high temperature [50]. The latter may have played a role in the poor survival of aphids exposed to the 39°C heat shock. Such effects could potentially influence the prevalence of symbionts in natural populations of aphids, though we again note that temperatures in that range are rare in central Europe, and the high capacity for dispersal in aphids would mitigate this. Symbionts lost during a spout of extreme temperature may be replenished quickly from regions that had not experienced such intensity.

Defensive endosymbionts may lose part of their protective effect against parasitoids under heat stress [17], [24], but they may still be beneficial in coping with such stress [21], [50]. However, there was little evidence for such an effect in this study. The 39°C heat shock was very detrimental for all aphid sublines, whereas the 35°C heat shock was not, and the *H. defensa*-free subline we used did not have a shorter lifespan or generally lower reproductive output compared to the infected sublines after exposure to 35°C, as could have been expected if the bacterium did provide such benefits. Nevertheless, we did observe a significant *G x E* interaction on lifetime reproduction, which was mainly caused by the subline 407^H402^ producing more offspring after the 35°C heat shock than in the control treatment (Fig. 3B). This observation was mirrored by an increase in lifespan of this line when exposed to 35°C (Fig. 3A), although that interaction was non-significant (Table 2). It was the same subline that showed increased parasitism by two of the parasitoid genotypes after exposure to 35°C (Fig. 2). It is thus tempting to speculate that *H. defensa* isolate 402 is more susceptible to high temperatures than the other two isolates and gets decimated by the heat shock, considering that *H. defensa* generally has a negative effect on *A. fabae*'s lifespan in the absence of parasitoids [43], [51]. In the present experiment, the difference between the uninfected line and the lines harbouring *H. defensa* was more pronounced for longevity than for lifetime reproduction in the control treatment (compare Figs. 3A & 3B), suggesting that the known life-shortening effect of *H. defensa* only came into play after most offspring had already been produced. More generally, by not obviously benefitting aphids under heat stress, *A. fabae*/*H. defensa* differs from the best-studied system that the symbiont is harboured by, the pea aphid *Acyrthosiphon pisum*, in which *H. defensa* has been shown to assist in the tolerance of heat shock [21].

As in Cayetano & Vorburger 2013 [26], in which the same basic experiment was conducted but with average temperature variation using constant regimes of 15°C, 22°C and 29°C instead of heat shock, the current study exposed the aphids but not the parasitoids to the temperature manipulation in order to avoid confounding heat shock effects on host resistance with effects on wasp behaviour. The absence of studies looking at the effects of temperature on the behavior of *L. fabarum* makes it difficult to judge whether it is likely to be an important component in the ecology and evolution of this system, though temperature-related stress can be important in influencing parasitoid behavior in other species [52], [53]. Similarly, sensitivity to the presence of *H. defensa* in potential hosts has not been verified for *L. fabarum*, but has been confirmed in other parasitoids [54], [55]. If heat reduces symbiont-conferred resistance, for which there is good evidence from pea aphids [17], [24] and some limited support from the present study on black bean aphids, parasitoids could potentially benefit, but only if they are not incapacitated by heat themselves. Parasitoids cannot benefit if high temperature harms aphids to the extent that they become unsuitable as hosts (as in our 39°C heat shock treatment). In this case, there should even be selection on parasitoids to recognize and avoid such hosts, though this may be precluded as an optimal strategy depending upon the costs involved [56], [57].

Overall, it seems plausible to suppose that under the range of conditions likely to be encountered naturally by this system, heat spikes are at most of limited importance in determining the outcomes of host/symbiont – parasitoid interactions. Nevertheless, we did gain some important insights. We found that endosymbiont defensive function can be maintained after exposure to heat shock at 35°C, at least for several combinations of host endosymbiont and parasitoid genotypes, and we found that despite some indication of a *G x G x E* interaction, the susceptibility rankings of aphid/endosymbiont sublines to different parasitoid genotypes remain remarkably robust over a large temperature range. Together with similar findings pertaining to average temperature variation in Cayetano & Vorburger 2013 [26], these results suggest that temperature may not in itself be an important determinant of the outcomes of *A. fabae* – *L. fabarum* interactions, a situation that should facilitate biological control programmes aimed at managing this and related systems.
